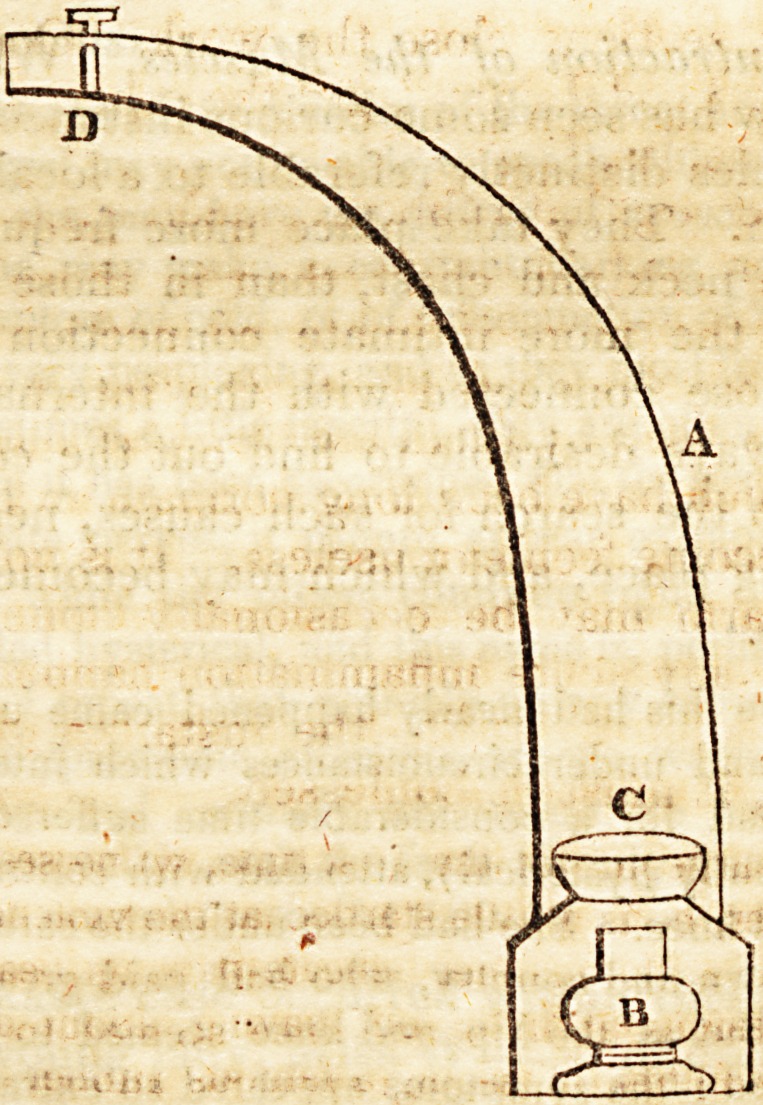# Farther Observations on the Lateral or Serpentine Curvature of the Spine, and on the Treatment of Contracted Limbs

**Published:** 1825-10-01

**Authors:** 


*Further Observations on the Lateral or Serpentine Curvature of the Spine, and on the Treatment of Contracted Limbs. With an Enquiry into the Effects of various Exercises, and other Means which are used to Prevent or Cure these Deformities: being a Supplement to the Work on Distortions of the Spine and Bones of the Chest.* By JOHN SHAW, Surgeon, and Lecturer on Anatomy. 8vo, pp. 195. Several Woodcuts. Longman and Co. London, May, 1825.

MR. SHAW has now become good authority on spinal diseases —and this class of human maladies is decidedly the prevailing or *fashionable* epidemic of the present period. We do not use the epithet *fashionable* with any idea of ridicule or satire; but because the word is strictly applicable to the complaint. A *fashionable* education has brought with it a *fashionable* shape and constitution—that is, a crooked spine, or a prominent hip, or a higher and a lower shoulder—an anterior and posterior (instead of the old fashioned right and left) breast, with many other *eccentric* modifications of form! But if these were the only evils attendant on modern education, they would surely be compensated by the intellectual attainments which every where meet the eye. The charms of music, painting, poetry, are far superior to corporeal charms—

> 'Tis not a set of features, or complexion,
> Or symmetry of form, that we admire.

If beauty soon becomes "familiar to the sight," so does deformity; whereas the accomplishments of the mind are, or ought to be, unfading and unchangeable. Such, at least, must be the opinion of those who direct the education of youth, and especially of the female sex. Unfortunately, however, we find that, in this world at least, matter clings to mind with astonishing tenacity. Bad health will ultimately sour the sweetest disposition—or, to come more immediately to the point, we aver that a crooked spine bids fair to produce a crabbed temper, however well stored may be the mind, and however accomplished the person. The world should ponder on these things, and consider whether they are justified in bartering "health of body, peace of mind," for a few shining endowments, that rarely contribute, after all, to domestic happiness in the station of wife and mother, however they may tend to shew off the possessor in the quality of maid or mistress. It is highly probable that this evil, like most others, will tend, in time, to remedy itself. The parent who hopes to get his daughter off in the world by her excessive education and accomplishments, may, perchance, often have those hopes blasted by the chilling hand of disease, which, while it deforms the body, will, most assuredly, deteriorate that mind, on which so much time, and labour, and expence, have been lavished—injuriously lavished! A time, we think, will come, when more attention will be paid to the *physique*, and less to the *morale* of the female sex. This desirable epoch will be hastened by the publication of works like that under review, because they necessarily circulate more among the non-professional portions of society than most other kinds of medical writings.

In the present work of Mr. Shaw, there is, unavoidably we believe, some repetition from the preceding work of the same author, occasioned by his following up the inquiry, "and going more minutely than formerly into certain subjects." He commences with some remarks on the appearances produced by lateral or serpentine curvature of the spine, in the following manner.

"When the spine of a girl about the age of twelve or thirteen is becoming crooked, the attention of the mother or governess is at first attracted by the state of the shoulders or breasts: at this age, indeed, most frequently by the latter; one breast either appearing larger than the other, or growing so unequally as to lead to a suspicion that it is diseased, or that 'one of the breast-bones is growing out of its place.' But, in a younger girl, the shoulders attract attention first, as the right appears enlarged, and, when the shoulder-blades are compared, the right is generally found farther removed from the spine than the left, and with its inferior angle lying flat upon the ribs, while that of the left projects.

"When the shoulders are thus affected, there is almost invariably a curve at the loins on the left side, which causes an apparent enlargement of the left hip; and it is in consequence of this, that a mother describes the state of her child, when the spine is slightly distorted, as 'a *growing out* of the right shoulder, and of the left hip.'

"When a girl so affected is in certain positions, one leg appears shorter than the other; when she walks, there is not only a constrained position of the head and neck, and an inclination to one side, but there is also an inequality in the step, so that the body is carried obliquely forwards, or with one side rather more advanced than the other. It may be frequently observed, that girls in this condition have a habit of putting one arm behind the back, and taking hold of the inside of the other elbow, thus assisting to balance the figure, by pulling down one shoulder and elevating the other." 3.

If the spine be now examined it will be found nearly in the form of an italic *f*, and perhaps with a slight bend outwards. The whole of the right side will be of a rounded or barrel-like form, while the left is diminished and contracted, the ribs being closer together than is natural. There will also be a depression or sinking in of the right, and a fulness between the ribs and hip of the left side. If the girl hold both arms above her head, the difference in the shape of the two sides will be more distinctly marked—and when the arms are brought down close to the sides, we may see between the left side and arm, but not between the corresponding parts on the right.

The alteration in the state of the shoulders being one of the first symptoms of deformity that are observed, some late writers, Mr. Shaw thinks, have been led into error in supposing that the dorsal part of the spine is the first distorted, and that of the loins the last. In common lateral curvature, it is Mr. Shaw's opinion that the curve at the loins is not only the first formed, but that it is the cause of the other curvatures higher up. By taking this view of the formation of distortion, Mr. Shaw has been led to attend more to the means of remedying the curve at the loins than that at the shoulders, and avers that he has found by experience that he is practically right—"for the only instances where the amendment of the curve between the shoulders has not followed the removal of the bend at the loins, have been where the upper ribs were much mis-shapen, or where anchylosis had taken place between two or three of the dorsal vertebræ."

We shall here introduce a rather long note, on the subject of the etiology of lateral curvature, particularly as regards the muscles of the spine.

"In the preceding volumes, I have examined several of the opinions which have been offered on the condition of the muscles of the spine in cases of distortion, and by reference to facts, both of natural and morbid anatomy, have shewn that many of them are unfounded: I shall here offer a few observations on an opinion pervading the works of all who have lately written on this subject, which, from being often acted upon, becomes of importance.

"It is conceived that distortion takes place in consequence of the muscles on the concave side of the curve, being increased in strength, while the power of those on the convex side is diminished. The shortening of the muscular fibres on the concave side is taken as the proof of their being increased in power, and also of their having been spasmodically or morbidly contracted.

"Any deviation to one side gives to the muscles fixed to the transverse and spinous processes of the concave side of the curve, increased contraction, whilst a corresponding state of relaxation or extension takes place in those of the opposite side; the muscles on the concave side acquire, comparatively, increased power, whilst those on the convex become proportionately debilitated, and the balance by which the spine is preserved in its erect form is necessarily destroyed.'—Observations on Distortions of the Spine, by W. T. Ward, p. 25.

"I have, on the contrary, shown that the muscles on the concave side are not the strongest. (See p. 68, in the octavo volume.) The idea that the muscles on the concave side are more powerful, and more contractile, than those on the convex part of the curve, seems to have arisen from the *consequences* of distortion having been mistaken for the *cause*. If the two ends of a muscle are approximated, the muscle gradually becomes shorter; a variety of proofs of this might be offered. A case in point, and in illustration of Mr. Hunter's views on the subject of contractility of muscles, is related in the Philosophical Transactions. A man broke his arm, the bones were not reduced, so that, when the man recovered, the bone was diminished to almost half its natural length. 'Some years after this accident, the person died. The biceps muscle of both arms was carefully dissected out, and, being measured, the one was found to be eleven inches long, the other only five, so that the muscle of the fractured arm had lost six inches, which is more than the half of its original length.' Similar effects are produced on the muscles of the fore part of the neck after burns, which pucker the skin so as to draw the chin towards the breast; and exactly the same thing takes place, not only in the small, but also in the large muscles of the back, and even in those of the lateral part of the abdomen, when their attachments are approximated by the yielding of the lower part of the vertebral column to the superincumbent weight.

"This shortened state of the muscles has been called a disposition to contraction, a kind of spasm which pulls the bones towards each other. But we have no proof that the muscles of the back, are ever affected in this way, in cases of distortion. It is, perhaps, incorrect to call it a *growth*, as there is actual diminution in bulk. But the examples that are offered may entitle us to conclude, that the shortening of the muscle is the natural result of the approximation of its two extremities.

"This is not to be viewed as only a curious question in physiology, but as one of much importance in the treatment of distortions of the spine and contractions of the limbs; for it will be found, that if the bones can be kept at a certain distance from each other, the shortened muscles will be lengthened. It is owing to their ignorance of this fact, that rubbers and shampooers are not so successful in remedying contracted limbs as they might be.

"After a description which is given of the curves of the spine, in the next page of the work from which I have quoted, it is stated, 'The *intertransversales muscles* of the concave side of each curve respectively would become contracted; those of the convex side of the curve, on the contrary, being in their extended state, would become smaller in size, and, consequently, weaker, so that if the weight were suddenly abstracted, they would no longer have the power of replacing or preserving the bones in their natural position, so as to bear the superincumbent weight; and, as every increased deviation from the perpendicular line would render the muscular parts still less capable of acting, the alteration of form, unless some means were used to counteract it, would become perpetuated.'

"I shall not deny the existence of the *intertransversales*, as they are in the list of the muscles of the back; but as, on actual dissection, we find them to be little more than mere membranous shreds, covering the membrana intertransversalis (particularly between the dorsal vertebræ); and when we consider, that one sacro lumbalis muscle is some hundred times more powerful than all the intertransversales that are enumerated, we cannot believe that three or four of these small muscles have any effect either in producing distortion or in preventing its cure. That the effect of distortion on the fibres of the intertransversales has been mistaken for its *cause*, may be exemplified by the state of the œsophagus in cases of complete serpentine curvature, or of hump back; for as the œsophagus, in these cases, does not follow the curves, but passes directly across, it is so contracted, that we might as well say that it is by an irregular action of the muscular coat of the œsophagus (which is at least as strong as the fibres of the intertransversales,) that the head is pulled towards the stomach, as that the spine is pulled from side to side by these small muscles; both opinions would be equally supported: the fibres of the intertransversales are found shortened, so are those of the œsophagus.

"I shall here repeat what I have attempted to prove in the preceding volumes—that, (notwithstanding the doctrines generally received), in the *common, lateral, or serpentine curvature*, the muscles do *not produce* the distortion, but become altered in form *in consequence* of the distortion." 13.

In the second section of Mr. Shaw's work he takes up the subject of etiology, especially in respect to the effects which the habits of sitting or lying in a particular manner have on the figure of a young person. He does not add much, however, to what he advanced previously, in his larger publication. Our author comes to the conclusion that, although mal-position, whether by day or by night, may *increase* a curvature of the spine that has already commenced—yet, that such cause will not *produce* the said curvature, if there be no other causes acting, and if the young person take proper exercise.

"The more I see of this serpentine curvature of the spine, the more I am convinced, that although the distortion will be always much increased, and occasionally produced by certain positions, it is generally caused, in the first instance, by the yielding of the lumbar portion of the spine to the superincumbent weight." 26.

The important question then naturally presents itself—what is it that causes this portion of the spine to yield?—"Is distortion produced by bad health?"—Some kinds of deformity, Mr. S. thinks, are produced by disease; but many circumstances, he adds, might be offered to shew that the lateral (or more properly speaking, *serpentine)* curvature of the spine seldom proceeds from this cause.

"Its frequent occurrence among girls who scarcely ever had a day's illness, is strong evidence of this. We may also adduce the fact, that although the poor, in large towns, are subject to various diseases of the spine, yet, in that class, the description of lateral distortion which is so frequent among young ladies is rarely seen; and, when it does occur among the poor, it is generally accompanied with some acute disease of the vertebræ, or is, to a great extent, combined with rickets, or a distinct scrophulous affection of one of the limbs." 27.

An important and difficult question in practice is, to distinguish between the cases where a scrofulous taint in the constitution is the predisposing cause of distortion, and those in which the curvature of the spine is solely owing to fortuitous circumstances.

"Why are the females in warm climates seldom affected with lateral curvature?"—The difference cannot, Mr. Shaw argues, be attributable to the mutability of our climate, since there are few examples of this curvature among the poor or the peasantry of this country, who are so much exposed to atmospherical vicissitudes. Neither can the ladies of the sun owe their straight shapes to tight lacings—for the looseness of their robes is well known. Their exemption from deformity cannot be owing to exercise—for they are proverbially indolent. Our author, therefore, is led to suspect, and not without reason, that there are some circumstances in the habits of life of the ladies of hot countries that would be beneficial in those of cold. But a system of indolence is unnatural to the latter, and, if pursued, would not answer.

"The question is now narrowed, and we are at liberty to enquire whether young ladies in this country have the advantages which nature dictates; if it can be shown that they have not, it will be no longer surprising that they should lose their natural form.

"It will probably appear that, owing to the prevalence of erroneous opinions on the question of exercise and rest, there is seldom a proper balance kept up between them. It is, perhaps, correct to say, that the less exercise a child takes, the more does it require general muscular relaxation in the recumbent position, and that the lighter and more sedentary the pursuits are, the more necessity will there be, either for active exercise or general relaxation. Thus in warm climates, where active exercises cannot be taken, the due relation of parts, or balance of the system, is preserved by great indulgence in the recumbent position.

"If we consider the manner in which young ladies are brought up, from the age of ten to sixteen, and keep this principle in view, we shall perhaps be able to discover the cause why they are more frequently deformed in a particular manner than those of any other climate, or even than the poorer classes in their own country.

"As long as a child continues in a state of nature, that is, while it is permitted to run freely about, and before it arrives at that age when the parent is induced to pay particular attention to its figure, the form is fine and perfect; but, about the age of nine or ten, what may often be truly called its miseries commence. Education is seriously begun, and the girl is no longer permitted to indulge in that playfulness which is not objected to in boys; indeed, it often happens that the first lesson a young lady receives, is an admonition that she is not a boy: when she walks, or when she sits, particular attention is paid to her manner, and the point most generally insisted on is, that she shall keep herself quite erect. For this purpose, or to give the chest a certain form, she is incased in a pair of stiff stays. Girls are thus early put under restraints not natural to their age. This, in some degree, renders them artificial, which is increased by the restrictions which are unavoidable in the acquirement of certain necessary accomplishments.

"If such habits be unnatural to the time of life, we cannot wonder that there should be a deviation from the natural growth of parts. It is not extraordinary that a child has its bowels disordered when its natural diet is changed; but we are apt to think it strange that the figure should not continue to grow as well *when we take great care of it*, as when the child was romping, and when no attention whatever was paid to its form. To set the bowels right, a variety of family recipes are often given, while the diet is neglected; but they are as ineffectual in restoring the natural tone of the digestive organs, as the staymaker's contrivances are in mending the shape. In both instances we endeavour to overcome nature, or to set it right by artifices, and often by artifices that are ill calculated for the purpose.

"Perhaps the reader is now prepared to admit the following view of the causes of the common slight curvature, when it occurs in a girl who, although not of a bad constitution, is listless, easily fatigued, and unwilling to take active exercise. The first cause which I would assign is the want of sufficient general exercise, and especially of that which acts more immediately on the muscles of the back; the second, on the almost necessary yielding of the lumbar portion* of the spine to the weight of the upper part of the body, if the girl be allowed to sit at work, or practise at the piano for hours without any artificial support; the third cause I would name is the habit of lounging or balancing the body on one leg; the fourth, the habit of sitting awry while writing or

---

"* This is the most moveable part of the spine, and although it supports the weight of the chest, head, and arms, it is not strengthened by the locking of its processes, nor by the attachments of the ribs, as the dorsal part is. As it is thus so dependant on its muscles, it must yield more readily than any other part when a girl is in a slightly debilitated state, either after recovering from fever or measles, or from the bad health that often accompanies a change in the constitution."

drawing; the fifth, the habit of sleeping on a soft bed and with a high pillow; the sixth, the more frequent use of the right than of the left arm; and, lastly, I would assign as a cause of curvature, most of the attempts that are made to correct the figure or to model it into a certain form. As so many of the means employed for this purpose, and for counteracting what are considered the disposing causes to distortion, frequently increase and even produce the curvature, it may be useful to endeavour to exhibit these effects. I am, therefore, confident that, to those who are interested in this enquiry, no apology is necessary for going, at some length, into the consideration of the USE OF THE INCLINED PLANE; of the UTILITY OF STAYS AND SIMILAR CONTRIVANCES; of the MANNER OF SITTING; of the MEANS GENERALLY EMPLOYED WITH THE INTENTION OF PREVENTING OR CURING A STOOP; and of THE EFFECTS WHICH CERTAIN EXERCISES PRODUCE ON THE FORM." 38.

The next subject discussed is that of the inclined plane. This having been found useful in the *cure* of spines a little twisted, is now considered a mean of prevention. This, Mr. Shaw observes, is a mistaken view. It is ridiculous to make a girl lie in a particular posture for an hour or two every day—while the position during eight or nine hours in the night is totally neglected.

"Is there any thing specific in the board, or is it more than the medium of affording rest; or is there any difference between the manner of lying on a board and that of lying on a mattress? Lying on a board is certainly more uncomfortable; and perhaps this is the reason why it is considered useful; for it seems to be an axiom, that if any thing connected with surgery is agreeable, it cannot be salutary." 41.

The inclined plane, in fact, is only good, as it gives rest and support to the body, and, viewed in this light, there is no good reason why it should not be made comparatively comfortable, as by cushions, for example, so that the patient may recline on it and be at ease, after a walk or a fatiguing lesson—times when the spine of a young person requires to be carefully managed.*

In respect to the night, our author adds little or nothing to what he has said in a former volume. The patient should sleep on a firm mattress, with little or no elevation of the head.

The practice of confining patients for months and years, in the horizontal posture, has been founded on the idea that the distortion depends on an undue contraction of certain muscles

* "It cannot," says Mr. Shaw, "be too often repeated, that the spine of the most delicate girl, if not already distorted, will not suffer by the erect position, as long as she is not fatigued, and continues in activity and exertion; it is during a state of lassitude and relaxation of the muscles that the horizontal position is necessary, for it is then that the bones and ligaments are in danger of yielding." 41.

of the spine, and on a diseased state of the vertebræ—an idea, observes Mr. Shaw, "completely erroneous." The bad effects of such a mode of treatment are gradually becoming more evident—and the use of the inclined plane is quickly falling into disrepute. Mr. Shaw next relates examples of the bad effects of the practice, for which we refer to the volume. He observes that it is an erroneous opinion that the spine becomes fixed at a certain age—a deceptive hope which has induced many a poor girl to confine herself to the plane for years. "Every one must have met with instances where the distortion of the spine has increased after the age of twenty-one." The plan, therefore, he thinks, is to be reprobated in cases of lateral curvature, as it tends to increase rather than diminish the curvature and the original causes of distortion. It almost invariably injures the general health.

Mr. Shaw next reverts to a subject discussed in his other work, namely, the question whether or not the pain, which is usually felt in some part of the spine, in lateral curvature, depends on a disease of the bones, or inflammation of the ligaments—or is it merely the consequence of certain causes that may be easily removed? Since his other work was published, he has seen several patients who have suffered severely from a mistake respecting the nature and cause of the pain. One instance was that of a young lady who had been confined nearly a year to the horizontal position, in consequence of a dull weary pain in the lumbar region of the spine, which was considered symptomatic of incipient inflammation or disease of bone. The said posture, however, produced no relief, and, at the end of four months, she felt the same pain when she attempted to walk. Still the complaint was considered inflammation, and six more months of confinement were enjoined, without benefit. Mr. Shaw was then consulted, and, on careful examination of the spine, became convinced that there never had been disease of the vertebræ. A different plan was adopted, and the young lady soon recovered.

"But, notwithstanding the issue of this case, it would be a more serious error to mistake the pain consequent on inflammation of the bones, for that which is usually attendant on lateral distortion."

Mr. Shaw gives us but little assistance in distinguishing between these two conditions. He observes that, when the vertebræ are actually diseased, there is generally a train of symptoms, in addition to the pain, which will assist us in discovering the nature of the case. Painful sensations in the back may depend on a variety of causes—as from pressure on some of the viscera in a great degree of distortion—on compression of some of the spinal nerves by one of the spinous processes themselves, &c.

Finally, Mr. Shaw's strictures on the inclined plane are only intended as objections to its being considered as the sole remedy, and to its unremitted use for months. He thinks the inclined plane essential to the cure of lateral curvature.

"And there cannot be a doubt that a young and delicate girl may be much the better of occasionally lying down; when she is growing quickly, and is, at the same time, delicate, the weight of the upper part of the body is obviously so much more than the lumbar vertebræ can support beyond a certain time, that common sense dictates the necessity of giving occasional ease and rest to the muscles of the spine, and this cannot be more effectually or more easily done than by lying down either on the inclined plane, or on a couch, where she may have room, while in the horizontal position, to shift her body as she pleases.*" 65.

OF THE PLANE AS A MEANS OF STRETCHING THE SPINE.

Machines, Mr. Shaw observes, have been used under the idea that, if the vertebræ could be kept separated from each other, the distortion would be removed. But, as they were generally made of iron, and attached to the body, they were often injurious. Then the plan of swinging the body by straps attached to the head, came into fashion, but was soon abandoned from its inutility. Confinement to the inclined plane was then said to be the only effectual means of curing distortion. As this was also found to fail very frequently, an apparatus was invented that was supposed to effect the forcible stretching of the spine, while the patient was stretched upon the plane. It is used in Paris, in an institution called the ORTHOPEDIQUE.

"* The bad effects of lying constantly in one position on the inclined plane, are so much obviated by friction over the muscles of the back, that a slight curvature may be cured in this way. But why do those who lay a patient on a board for months, with the view of subduing an inflammation of the bones, and restoring the equilibrium of the actions of the muscles, recommend the spine and adjacent parts to be rubbed and shampooed for two hours during the day? Is it not contrary to their theory of the causes of distortion, and to their views of the mode of curing curvatures? But, so far am I from objecting to the combination of friction with rest, that I consider it as the only means of saving patients, who are laid on the plane, from falling into the most miserable state of debility. Since friction and shampooing are only a form of exercise, why not carry the system of combining exercises with the confinement a little farther, and especially if it can be done with safety? It is cruel to imprison a young lady for months, if all the advantages of lying on the plane may be attained, and the disadvantages avoided, without making her undergo such severe discipline as the constant confinement to one position must be."

By this invention, patients are debarred from taking exercise, and, therefore, it is evident that it must be even more injurious than the collars which have been in use to support the spine.

"A most untenable theory still prevails in this country; that distortion is produced by dislocation of the vertebræ; and, upon this theory, a practice has been founded which is not only inefficacious, but so dangerous as to render it a matter of duty to take every opportunity of exposing it. The practice alluded to, is the attempt, by main force, to replace vertebræ said to be dislocated, and to keep them in what the *operator calls their proper position* by bandages, heavy weights, and confinement of the patient to the horizontal posture. But the ideas on which this plan of treatment has been founded, are so diametrically opposite to what we should deduce from the facts of anatomy, as to be scarcely worthy of a serious refutation. We have only to compare the sketch below, with the description given of this mode of practice, to learn that the idea of reducing dislocated vertebræ to cure distortion, is not new, but adopted from the suggestion of ancient authors, who, although worthy of credit in many respects, must be admitted by all who are conversant with anatomy, to have occasionally dealt in the fabulous.* The danger to which the spinal marrow would be subjected, by any forcible attempt to shift one of the bones of the spine, must be obvious to the merest tyro in anatomy.

"This is copied from the chapter in Ambrose Parée, on the cure of the projecting vertebræ.

"* In Hildanus, under the head of Dangerous Ignorance of a Barber Surgeon, '*Perniciosa Tonsoris inscititia*,' a case is given where the operator attempted to replace two projecting vertebræ, by laying the patient on his face, and then pressing on the bones with both his knees. The horrible effects produced are well described; but, in the next page, a case is given, where Hildanus says the vertebræ were dislocated by the devil!"

"The modern operator thus describes the method:—

"'I caused the spine to be stretched daily, for an hour at a time, in order to draw out, and, in some degree, to separate the vertebræ from each other. This operation was performed by means of the shoulders being pulled by one person placed behind the head, and the feet, at the same time, by another, in opposite directions, the Colonel all the while lying on his back. During the period that this process was going on, I continued to make, with my own hands, firm pressure upon the sternal ends of the ribs, first on the one side and then on the other. By this contrivance, they were forced to act powerfully at the other end, upon the depressed vertebræ. This was done to drive them outwards, and towards their proper situation in the column.'—Medical and Physical Journal, vol. 44.

"It may appear almost incredible, that such an attempt was ever made in this country; but we have the best evidence of its having been done, that of the operator himself, and I can state that a mode nearly similar, although even more frightful, has been practised in London by the same operator within these twelve months. I have been consulted by several patients who had not only been rigidly confined to the horizontal position for two years, but had submitted, almost daily, during that time, to be laid for an hour on their face, to have the feet fastened to one end of a bed frame, while a cord that was attached to a strap round the back of the head, was wound up by a windlass that was fixed to the other, and which, from its appearance, seemed to be of sufficient power to raise half a ton. I was told that while the spine was thus stretched, the physician tried, with a small wooden instrument, to *push in the dislocated bones!* Cases, detailed by this operator, to show the *wonderful* effects of this plan of practice, may be found in the 44th and 45th vol. of the London Medical Journal." 72.

*Stays and other Artificial Supports.* The general impression is, that stays are hurtful. Some, however, are of a contrary opinion. Our author asserts, somewhat paradoxically, that stiff stays may produce distortion, and yet that those which are generally worn are not stiff enough. It is unnecessary to say, that no other stays or supports for the back could ever have been designed by Nature than the muscles of the spine. Every artificial aid, in the way of stays, must, consequently, weaken the muscles, by depriving them of their natural and due action. Still, all our arguments will not change the fashion of the day. We may possibly modify the injurious customs around us. Stays should not be put on, Mr. Shaw thinks, till the age of ten or twelve at least, since even fashion does not require other than natural shape in a child.

"When stays *must* be put on, they should be loosely laced, for the tighter they are, the more do they act as compressing bandages, which not only prevent the natural play of the muscles, and thus weaken them, but even waste and lessen their size. That such may be the effect of pressure is often seen in the wasted leg of the mendicant, which, through tight bandaging alone, can be reduced to that condition which excites our commiseration.

"If stays are put loosely on, and only worn occasionally; and if the girl takes sufficient active exercise, and rests in a proper manner when fatigued, there is little danger of the form suffering even from strong stays. Bnt although by this method, stays may be rendered almost harmless, there will be some difficulty in pursuing it, as the girl will feel the occasional bondage very uncomfortable. The annoyance produced by it, is marked by the flushing of the face from impeded respiration, and by a stiff and constrained manner of walking. The remedy generally proposed is, that *she should wear the stays until she gets used to them*; this advice will probably be followed; and then it is likely that the bad effects, already described, will ensue." 83.

But it is still to be shewn that stays may often be necessary—and that those generally used may, under certain circumstances, not be stiff enough. It is necessary to remark, that a girl's education weakens the muscles of her back, as well as other muscles in the body; yet she is expected to be able to keep her spine as erect as if she had the strength of a porter. There is a mistaken notion respecting the effect of stays not stiffened with bones. Mothers think their children in no danger of becoming distorted, by wearing such stays, forgetting that the tight bandaging of the chest, when persisted in, is more injurious than the effect produced by stays which support the figure, even to the degree of obviating the necessity for muscular action.

"It is true that a bandage, occasionally applied, gives support and strength; but if constantly worn, it produces a wasting of the part. Proceeding on this view, it may be stated that if tight stays *must* be worn, they should be made sufficiently stiff and strong to sustain the weight which the muscles that have become deteriorated by want of action are unable to support. If the stays are not made so, (the muscles or natural means of support being already weakened) there is danger of certain ligaments of the spine yielding, and hence, of the vertebræ falling out of their natural line, and thus producing curvature of the whole column. But so prevalent is the persuasion that stays are injurious, and so little does the principle, on which they are useful or hurtful, seem to be understood, that the first thing generally recommended in a case of weakness, or yielding of the spine, is that the stays should be thrown aside, or at least that all the bones should be taken out. If a determined plan of practice, combining appropriate exercises with rest and proper support, is to be pursued, this may be all very well; but when a girl is weak, to deprive her of her artificial supports, and to leave her at once to her own physical resources, seems to be acting in a manner very much at variance with the dictates of common sense. I have seen so many instances of the bad effects of this plan, that I cannot help expressing myself strongly." 85.

We think the above view of the case is most unquestionably the correct one. There is no doubt that, however tortuous is the feeling of tight stays at first, the poor girl soon becomes not only accustomed to them, but, in a great measure, dependent on them for support—especially after any illness when she is scarcely able to sit up so long as to dress, so feeble and relaxed are the muscles of the back. A mother naturally takes alarm at this, but attributes it to the late illness. The girl continues in the same state, and probably complains of lassitude and a weary pain in the back. The spine is now examined, and perhaps a slight curvature is discovered, with pain on pressure of certain vertebræ. She may now be condemned to lie for a year or two on the inclined plane—at all events, the bones are ordered to be taken out of her stays, under the idea that, as they produced the distortion, the increase of the evil will be prevented by their removal. It is evident that this removal will only increase the malady—the muscles now being in want of support. "Although the supervention of distortion affords a good argument against the previous use of stays, they may nevertheless be necessary to a person in this state." When the spine yields, therefore, instead of throwing away the stays, we should make them stronger, so as, if possible, to take off the weight of the shoulders and head from the lower part of the spine. The stays will not cure a distorted spine, but they may tend to prevent the spine from becoming more crooked, especially if they be combined with means of strengthening the muscles.

*Of the Means of Curing a Stoop.* In slight distortions there is frequently a stoop, to cure which, some instrument is usually put on, by which the head and shoulders are held back. For the shoulders, the common back collar is applied—while, to hold back the head, a ribband is brought over the forehead and fastened to the collar. These means in use, the figure looks straight, though stiff and constrained—but the moment they are removed, the head and shoulders fall more forward than before their application. This is easily explained, by the wasting of the muscles from want of use. There are many other contrivances for the cure of a stoop, on the same principles, but all ineffectual. The plan of carrying a weight behind, appended by a ribband that goes round the forehead, is worse than the others, because it calls into action the anterior muscles of the neck, and renders the posterior ones passive, thereby increasing the evil it is intended to remedy.

"We have many opportunities of observing the incorrectness of the principle on which all similar plans for the cure of a stoop have been founded. For instance; porters who carry burthens on the back, by the assistance of a band round the forehead, always stoop; while those who carry baskets before them suspended by a band round the back of the neck, are peculiarly erect. But the most remarkable example of the effect from the head being pulled back by a weight hung behind, is the condition of the women who carry salt in the streets of Edinburgh, for they may be recognised as much by their miserable sardonic grin, which is caused by the constant excitement of the platysma myoides muscle, as by their stoop." 97.

Here Mr. Shaw relates the case of a gentleman who had, for many years, worn one of the collars invented by Mr. Chesher, and who had the muscles of the back so weakened by it, as to be rendered incapable of supporting the column, while those on the fore part of the neck were so increased in strength, by the constant resistance opposed to them by the strap passing from the suspending rod under the chin, that whenever the strap was loosened, the chin was forcibly drawn towards the chest. By this the windpipe was pressed down or almost doubled on itself, and a sense of suffocation produced, obliging him to throw himself on his back. With much difficulty Mr. Shaw persuaded the patient to place on his head about fourteen pounds of shot. He was much gratified to find that, instead of having his head weighed down by the shot, he could support it, and could breathe with ease while in the upright posture. The object of placing the weight on the head was to give an additional stimulus to the muscles on the back part of the neck, and thus excite their action. By combining a variety of exercises, and gradually diminishing the weight on the head, Mr. Shaw had soon the pleasure of seeing his patient walking and sitting in a state of great comfort, without any artificial support.

*Effects of Exercise.* The question of exercise in spinal distortions, has lately been much agitated. It is very true, as Mr. Shaw observes, that the desire to go to the extreme of every fashion extends its influence even to the education of children. But a few years ago, young ladies were kept in a very inactive state, and now they are called upon for such violent exertions as to threaten another danger of an opposite kind. Many of the feats of strength, says Mr. Shaw, which young ladies now perform, are fitted only for athletics, and as they are seldom proportioned either to their age or constitution, the shape may be injured, while the health can receive no benefit from them. The bad effects of working a young horse too early are well known. And so among the children of the poor, who are put upon tasks beyond their natural power, we see premature age produced. Even good and nourishing food is not sufficient to avert the consequences of too early over-exertion—especially if it be sudden, and entered upon without previous training. In respect to the effect of gymnastic exercises in improving the form, Mr. Shaw offers the following observations.

"We have frequent opportunities of observing the actual deformity which arises from the disproportionate development of any particular class of muscles. For example the legs of those opera dancers, who pride themselves on their powers of making extraordinary leaps and pirouettes, are almost herculean, while their arms are comparatively diminutive. Similar effects are produced on the form of those who practise horsemanship and tumbling, although these persons are sometimes better proportioned than the dancers, from their muscular system being more generally exercised; still their muscles are so unnaturally increased, by the violent exertions necessary to their feats of strength, that their appearance may also be considered as approaching to deformity.

"But there is a more important circumstance to be considered in regard to the propriety of making children use occasional violent exertion;—the changes which may be thus produced in the condition of the ligaments of the joints. When muscles are gradually increased in strength, the ligaments become strong in proportion; but the ligaments are as likely to be hurt from the muscles being suddenly called into violent action and at an early age as by any accidental twist or strain. They are in this way liable to become spongy and relaxed, so as to produce weakness, or a condition similar to that of the joints of a young horse which has been gallopped hard, or obliged to take great leaps before he has acquired his full strength; indeed, there is much resemblance in the condition of a joint with the ligaments strained, to that of a horse which is broken down or wind-galled."* 126.

Such consequences may be guarded against by gradually increasing the degree of exertion. But even here, the joints may be injured by the unnatural lengthening of certain ligaments, as in the ancles of opera dancers, &c.

It cannot be supposed that these observations are directed against properly regulated exercise, the utility of which is admitted, but against excesses. By exercise, the several parts of the body are fully developed, and it is only by its regular per-

"* Small bunyons or ganglions, which are similar to what the farrier calls wind-galls, are sometimes found about the ankle joints of delicate girls, who have over-excerted themselves in dancing."

formance that they are perfected, and preserved from falling into decay. In this climate, we cannot even enjoy the fresh air unless we, at the same time, keep our bodies in active exercise, and every one knows how much regular exercise conduces to the general activity of the frame, and the operations of the digestive organs. No other remedy is equal to it for correcting that morbid excess of nervous irritability which is too often observed even in early periods of life.

The question recurs—What are the appropriate exercises? It is with exercise as it is with food. Sanis omnia sana. To a boy in health, it is almost ridiculous to prescribe any particular kinds of exercise—but they may be highly useful to those who are naturally indolent. They are not, however, Mr. Shaw thinks, to be solely trusted to for the cure of spinal distortions. He has not seen any instance where exercise alone has cured the disease. It is impossible to lay down any particular plan of exercise, as it must be varied and adapted to each particular case. The reader, however, will find many judicious observations and hints in the work before us, from page 138 to page 169.

Two short sections close the work. One "On the Treatment of Contracted Joints," and the other "On Nervous Contraction of Muscles."

Mr. S. observes, that rubbers and shampooers will sometimes succeed, where expert surgeons fail. These operatives (if we may so call them) proceed in a much more violent manner than those who know the structure of the parts would venture upon. The measures used by them are well calculated to call into action parts which have been long dormant in function, and, consequently, become feeble or useless. It is no wonder, however, that some harm may be occasionally done by these violent means, when any acute inflammation happens to be going on, or set up in a joint. Still, the instances of injury from this plan are rare, it must be confessed.

"When a surgeon, for the first time, witnesses the operations of a professed rubber, he is a little startled at the violence of his operations, and is surprised at the manner delicate patients bear them. Such were my own impressions at first; but having, about eight years ago, had frequent opportunities of seeing a famous rubber at work, and having witnessed the result of his treatment in several cases, I was so satisfied that, if judiciously combined with other modes, it might not only be safe, but of the greatest use, that I have since been in the habit of ordering the women, whom I employ on these occasions, to rub and shampoo with a degree of violence, which, to some practitioners, might appear almost unwarrantable." 173.

Mr. Shaw is particularly careful in the application of means to prevent the contraction after the limb has been shampooed, by proper position in the night. These means are simple and obvious. If the limb be kept constantly encased in a machine, and no exercise permitted, the muscles will waste, and when the promised period of cure is completed, the limb will be found incapable of supporting the weight of the body. Hence, the system of shampooing is far preferable to that of the instruments, if only one system is to be pursued; but the judicious practitioner will combine them, and thus draw forth the beneficial effects of both.

"In the treatment of contracted limbs, the application of vapour is often of great service. There are many modes of using it, but perhaps the plan sketched below is one of the most convenient. It is an improvement on the hot air bath, which was first used in this country, at the Middlesex Hospital. I contrived it for a young lady who had contraction of the muscles of the hip and knee, and who was always much enervated when the whole body was exposed to the vapour, as in the baths at Brighton.

"A is the tube, about three feet long, that is commonly used for the hot air bath; a hole is to be cut in its side near the bottom, of sufficient size to admit a copper pot, C, about 2½ inches deep, and 3 in diameter. This pot is to be supported by wires passing across the tube, but leave a space of three-fourths of an inch all round; the opening is to be closed by a small door. B, a spirit lamp, or an Argand lamp, trimmed with cocoa-nut oil, may be used. D is a stop-cock; a piece of wood is to be nailed to the back of a chair (below the seat), and through this, the nozzel of the pipe, which may be about an inch and a half in diameter, is to be passed. The nozzel may be directed rather downwards; if the chair has not a cane bottom, the seat should be perforated with holes, which are to be covered with a piece of flannel. The patient may have either two flannel petticoats, or two pieces of calico tied round her waist, which falling down surround the chair.

"The pot C is to be filled with boiling water, and the spirit or Argand lamp is to be placed below it (plenty of air must be admitted by the side and under the lamp); the water is very rapidly converted into steam, which issues under the chair in the form of vapour; a quantity of heated air also rushes up, so that the body may be quickly exposed even to a greater heat than if it were acted on merely by vapour. The degree of heat may be easily regulated either by the stop-cock at D, or by altering the flame of the lamp. If it be an Argand, it may be lowered, if a spirit-lamp it must be covered up in part, or withdrawn.

"By such an apparatus (the expence of which is little more than a guinea, if the pipe be made of tin), a vapour bath sufficient for the whole body may be prepared in a bed-room in five minutes." 179.

*Nervous Contraction of the Muscles.* Within these few years, Mr. Shaw has seen some curious instances of contraction of certain muscles distinctly referable to a local affection of the nervous system. They take place more frequently in certain muscles of the neck and chest, than in those of the limbs—probably from the more intimate connection of the cervical nerves with those connected with the internal viscera. Although it is always desirable to find out the *causes* of things, we must not, in the search for such causes, neglect the *effects* which are taking place, and which may become irremediable if not attended to.

"A case where this had nearly happened came under my observation last spring, and under circumstances which interested me much. A young lady had for a considerable time suffered from a train of symptoms (apparently hysterical), attended with contraction of the muscles of the hip and knee. She had been under the care of several practitioners, both in town and country, who had paid great attention to her general health. But as the hip and knee seemed to get worse, a consultation was called; the symptoms appeared so extraordinary, that the senior consultant plainly said that the patient was deceiving us; but I opposed this opinion, because I considered it more likely that she might be suffering from a peculiar nervous affection which we could not comprehend, than that she should not only deceive her friends, but submit to much inconvenience and pain. I therefore proposed, that whatever might be the cause of her present condition, whether it was a trick or not, we should do what had not yet been done,—endeavour to counteract its effects,—for her ancle was at this time turned round, so that she walked on the out side of her foot, and the knee was bent outwards to such a degree, that the external lateral ligament was nearly an inch longer than natural; her spine was also becoming distorted.

From having expressed this opinion, the case was rather forced upon me; and as I saw that the gentlemen who were in consultation did not expect that I should succeed in making the patient better, I was naturally more anxious than usual about the result. In the course of a short time the limb was much improved by the use of the vapour bath, shampooing, rubbing, and artificial supports, and attention being, at the same time paid to the general health, the young lady became so much better, that now, (within twelve months) she can walk so that scarcely any lameness is apparent. I have no hesitation in asserting, that if the state of the ankle and knee had not been attended to, they would soon have been irrecoverably injured." 185.

Young women are often accused of feigning diseases, when the medical attendants are not able to account for the symptoms; and of these we have seen some curious examples. We were, therefore, much pleased with the following passage in the work before us.

"Nervous affections of the muscles of the throat are more common; as, for example, a girl may not be able to speak, although she can swallow with ease, or she may have the power of speech without that of swallowing; or she may make a constant barking noise without any accompanying symptom of cold, or of inflammation of the throat. Such cases are certainly very perplexing, and as they most frequently occur in girls who are sometimes a little fanciful, they are often suspected to be feigned. But since it has been ascertained that the several functions are regulated by separate nerves, it cannot appear extraordinary that one may be disturbed while the others continue right. Such cases have been generally called hysterical, and this name is, perhaps as applicable, as any other; but I repeat, that scarcely on any occasion, when a girl has symptoms of a spasmodic affection, are we warranted in alleging that she is feigning, and certainly not unless we can show that the symptoms are incompatible with the facts already discovered regarding the functions of the several nerves." 189.

A very remarkable case occurred within these few years, in the person of a young lady, Miss W——, of Stoke Newington, who was seen by a great number of physicians and surgeons of this metropolis—some of whom considered the complaint as simulated.

The young lady, after a walk in her garden in a damp and cold day, was seized with such difficulty of breathing and incessant cough, that her medical attendant supposed there was laryngeal inflammation, and immediately bled her, but with very little or no benefit. The breathing continued with such a loud croupy sound, that no person in the house could get any sleep, nor did the young lady herself sleep for many nights in succession. All this time, however, the pulse was quiet, the skin cool, and the tongue clean. One physician pronounced the disease ulceration of the larynx, and requested that he might be sent for when the lady died, as he intended, with leave of the relations, to open the body, and demonstrate the truth of his opinion. Other physicians thought differently—and one or two accused the patient of simulating these distressing symptoms. The complaint continued, with slight remissions, for nearly two years, and was once interrupted completely, for a time, by the carbonate of iron, in scruple doses, prescribed by Sir Astley Cooper. Tonics, on the whole, did more good than any other class of remedies. It is now more than two years since the commencement of this unfortunate young lady's sufferings. Her breathing is, at present, easy, but she has entirely lost her voice, not being able to make herself understood, even by whispers. We hope Mr. Wight or Mr. Richardson will one day state the particulars of this most curious and interesting case. The former of these gentlemen was requested to keep notes of it by the Editor of this Journal, who occasionally visited the patient. We consider it as a case corresponding with those alluded to by Mr. Shaw.

The spasmodic affections in question do not often occur in the limbs; yet Mr. Shaw has seen one case where the knee has continued for weeks as stiff as if an iron rod had been passed from the tibia to the centre of the femur. For this affection he repeatedly applied leeches and blisters, but would not do so again, "as such cases often get well suddenly, and do not appear to be influenced by local treatment."

"A young lady had a pain in her knee, the muscles of which were, at the same time, violently contracted. She had been for some months under the care of a surgeon in town, who applied blisters, &c. but without any good effect. She went down to Brighton, used the vapour bath, and was shampooed; in a few days afterwards she was seen walking on the Steyne.

"In such a case as this, we cannot wonder that the patient should be accused of deceit by the surgeon, or that Mahumed, the shampooer, should be considered, by the friends of the patient, as more clever than her surgeon." 193.

We must now conclude our short notice of Mr. Shaw's work. Our readers will have perceived that it consists more of strictures and remarks on doctrines and practices already in use, than of new doctrines or plans of treatment proposed by the author. Yet there are many original observations, and some novel views, physiological and pathological, scattered through the volume. Mr. Shaw's criticisms are generally just, and his book will be very useful in correcting erroneous notions, especially among non-professional readers. Those who have the superintendence of the education of female youth, would find in this volume most valuable hints for preserving the health as well as the symmetry of the young ladies under their charge. Mr. Shaw is already so favourably known to the profession as to insure a good reception for his work in that quarter.

## Figures and Tables

**Figure f1:**
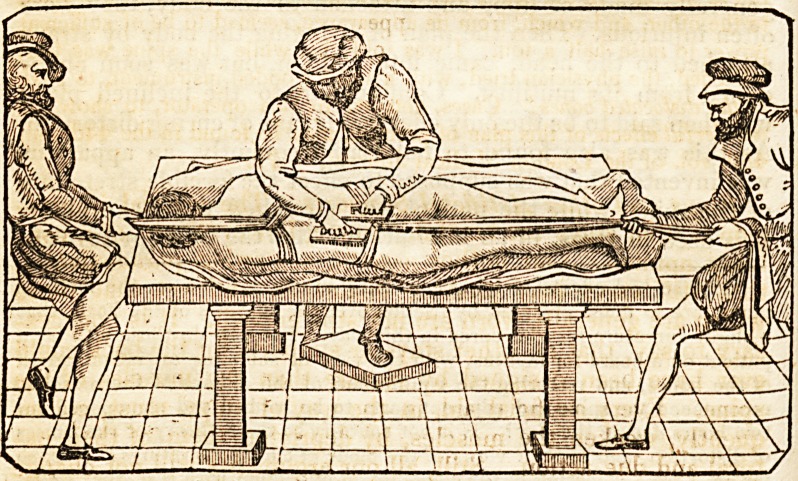


**Figure f2:**